# Scientific integrity and Nordic quality will remain the hallmark of *Acta Obstetricia et Gynecologica Scandinavica*

**DOI:** 10.1111/aogs.15026

**Published:** 2024-11-25

**Authors:** Ganesh Acharya

**Affiliations:** ^1^ Department of Clinical Science Intervention and Technology (CLINTEC) Karolinska Institutet Stockholm Sweden; ^2^ Center for Fetal Medicine Karolinska University Hospital Stockholm Sweden; ^3^ Women's Health and Perinatology Research Group UiT—The Arctic University of Norway Tromsø Norway

After serving 10 years, I will finish my term as the Chief Editor of *Acta Obstetricia et Gynecologica Scandinavica* (AOGS) in the end of December 2024. It gives me an immense pleasure to know that our more than a century old journal^1^, ^2^ is in very good condition and I am leaving it in the hands of an excellent team of editors, which will be led by Prof Amarnath Bhide from St. George's Hospital and City St. George's, University of London, UK, who has more than 15 years of editorial experience and has been a Deputy Chief Editor of AOGS for the last 6 years. It is a historical moment for our journal as Amar is the first Chief Editor of AOGS appointed from outside Scandinavia, emphasizing and reflecting the international spirit, authorship, and readership of the journal. Reaching beyond Scandinavia has been one of our priorities in the last decade, which has been only possible by the support of an International Editorial Board consisting of highly qualified experts in their respective fields, and I am very grateful to them all.

In my first editorial as the 12th Chief Editor of AOGS in January 2015 entitled “*AOGS: An international journal with Scandinavian quality*,” I wrote “…our focus will be on improving the quality of publications and their visibility, assuring ethical conduct in research, promoting dissemination of important findings and improving service to our authors.”^1^ A number of steps were taken to ensure quality and scientific integrity of published articles including a close collaboration with Chief Editors of other journals in our specialty.^3^, ^4^

What is the measure of quality of a peer‐reviewed medical journal can be debated, but there is no single acceptable indicator of quality. In my opinion, what are the aims of a journal and what does the society that owns it really stand for are as important as how often the journal articles are read and cited. AOGS is owned by the Nordic Federation of Societies of Obstetrics and Gynecology (NFOG), a professional nonprofit organization that aims to promote scientific collaboration among obstetricians and gynecologists in the Nordic countries and beyond to improve health and well‐being of girls and women globally. Shared attributes of Nordic quality are creativity/innovativeness, openness/transparency, compassion/equality, mutual respect and trust (social cohesion), and commitment to sustainable development. We have tried to assure the quality of AOGS sticking to Nordic values. Self‐discipline, involvement of the whole team in planning, implementation and governance supported by direct open communication, and regular educational activities have been crucial for continuously improving the quality of our journal.

The importance of responsible ethical conduct cannot be overemphasized in scientific research and publication. We have meticulously and systematically worked together with the authors (and their institutions when required), editors, reviewers, and publisher for over a decade to ensure that the ethical standards are met in all research articles published in AOGS.

One significant development that has happened recently is the 10th revision of World Medical Association (WMA) Declaration of Helsinki published on October 19, 2024, in JAMA.^5^ It has introduced some important changes that are likely to have a positive effect on how scientific research is conducted and published. Among other things, it includes statements on upholding ethical principles even in public health emergencies, safeguarding patients as well as healthy volunteers participating in research, requirement for adequate qualification, competency, and scientific integrity to conduct medical research involving human participants and avoiding any misconduct and research waste.

Good intentions do not always lead to desired practices or outcomes. A such example is the exclusion of pregnant people from participating in certain research studies, especially interventional clinical trials, due to the misinterpretation of WMA statement that vulnerable participants—such as pregnant people or members of ethnic and racial minorities—should be protected. However, findings of research studies carried out in less vulnerable groups, such as men or nonpregnant people, may not be applicable to pregnant people, and the harms of exclusion may be more than the harms caused by inclusion of pregnant people. Therefore, it is important that pregnant people are involved in research while ensuring their safety. This has been now recognized in the most recent revision of WMA Declaration of Helsinki.^5^ Hopefully, this will lead a more pregnant people participating in clinical trials substantially improving the current situation.

We are an international journal, and we strive for excellence. We promote fairness, openness, and equality. To the best my knowledge, our journal was the first society‐owned general obstetrics and gynecology journal to go fully open access. The transition to open access publishing model has been relatively smooth, and it has helped to disseminate and share good science worldwide providing unlimited access to the original research articles, reviews, and commentaries published in AOGS to our readers, which will continue to help to improve their scientific knowledge and promote evidence‐based clinical practice positively impacting women's health.

It has been an honor and privilege to serve as the Chief Editor of AOGS for a decade. I had an unconditional support from the NFOG Board, AOGS Editorial Board, and colleagues around the world. It was a satisfying experience to learn by doing things right as a team with your help rather than learning from my own or others’ mistakes or failures. I know there will be many challenges and opportunities in the future. Some posed by new technologies, such as the Artificial Intelligence, are already knocking the door.^6^ However, I truly believe that AOGS has a bright future ahead, and the scientific integrity and Nordic quality will remain its hallmark for years to come.
